# Lean back or lean in? Exploring social loafing in human–robot teams

**DOI:** 10.3389/frobt.2023.1249252

**Published:** 2023-10-18

**Authors:** Dietlind Helene Cymek, Anna Truckenbrodt, Linda Onnasch

**Affiliations:** Institute of Psychology and Ergonomics, Chair of Psychology of Action and Automation, Technische Universität Berlin, Berlin, Germany

**Keywords:** human–robot interaction, team effects, motivation, social loafing, quality control, sequential redundancy

## Abstract

**Introduction:** Thanks to technological advances, robots are now being used for a wide range of tasks in the workplace. They are often introduced as team partners to assist workers. This teaming is typically associated with positive effects on work performance and outcomes. However, little is known about whether typical performance-reducing effects that occur in human teams also occur in human–robot teams. For example, it is not clear whether social loafing, defined as reduced individual effort on a task performed in a team compared to a task performed alone, can also occur in human–robot teams.

**Methods:** We investigated this question in an experimental study in which participants worked on an industrial defect inspection task that required them to search for manufacturing defects on circuit boards. One group of participants worked on the task alone, while the other group worked with a robot team partner, receiving boards that had already been inspected by the robot. The robot was quite reliable and marked defects on the boards before handing them over to the human. However, it missed 5 defects. The dependent behavioural measures of interest were effort, operationalised as inspection time and area inspected on the board, and defect detection performance. In addition, subjects rated their subjective effort, performance, and perceived responsibility for the task.

**Results:** Participants in both groups inspected almost the entire board surface, took their time searching, and rated their subjective effort as high. However, participants working in a team with the robot found on average 3.3 defects. People working alone found significantly more defects on these 5 occasions–an average of 4.2.

**Discussion:** This suggests that participants may have searched the boards less attentively when working with a robot team partner. The participants in our study seemed to have maintained the motor effort to search the boards, but it appears that the search was carried out with less mental effort and less attention to the information being sampled. Changes in mental effort are much harder to measure, but need to be minimised to ensure good performance.

## 1 Introduction

Traditionally, robots have worked with little or no interaction with human colleagues for safety reasons. In the automotive sector, for example, the payload and speed of large single-arm robots handling body parts pose a serious risk to human workers. However, there is also an emerging trend to bring humans and robots closer together, both physically and temporally, offering a wealth of new applications (Restrepo et al., 2017). This structural shift from a separate workspace to a shared workspace with cooperative or collaborative facets resembles a paradigmatic change. While the human–robot relationship with conventional robots can be well described as a tool-operator relationship, the relationship with robots designed to work alongside humans increasingly resembles that of human teamwork, including its forms of interaction (Wiltshire et al., 2013; Lewis et al., 2018; Onnasch and Roesler, 2021). Examples of existing human–robot teams can be found in warehouses, where robots and humans work together to pick items for shipping, in complex final-assembly tasks in automotive manufacturing, or in quality control of manufactured goods. While such human–robot teaming can also help to compensate in sectors affected by a shortage of human labor (Wisskirchen et al., 2017), it is most often intended to increase the efficiency and ease of work for human workers (e.g., Lefeber et al., 2017; Neto et al., 2019). Moreover, some robots are specifically designed to complement human skills in order to optimize work outcomes (e.g., Wischmann, 2015). An example of such human–robot interaction (HRI) can be found in the increasingly digitized quality inspection of electronic components. Here, for example, robotic arms are used to scan welds and seams with profile sensors to detect cracks or other defects in the components (e.g., Brito et al., 2020). These systems are getting better and better, with powerful sensor technology that surpasses human vision, especially in terms of endurance, but sometimes also in terms of accuracy. Occasionally, however, these robotic vision systems can miss the finest cracks or mistake small grains of dust or oil residue for very fine cracks. These are conditions that humans can often distinguish relatively well. Using human–robot teams in a way that exploits the complementary strengths and skills of humans and robots therefore has great potential for optimizing work results in this case.

In addition, teamwork can improve work outcomes beyond simply combining complementary strengths. In human teams, where more than one person is responsible for completing a task, several positive effects on individual performance can occur. For example, people show increased levels of effort and performance when performing simple and well-trained tasks in the presence of others compared to when they are alone—a phenomenon called social facilitation (e.g., Triplett et al., 1898; Zajonc, 1965). Positive social-competition effects can also enhance performance in human teams, when individuals want to outperform each other on tasks where individual contributions to the task are recognizable (Stroebe et al., 2018). Such performance-enhancing team effects may also occur in human–robot teams, as it has been found that humans easily perceive computers as team partners (Nass et al., 1996) and tend to apply social rules, expectations, and behavioral patterns from human interaction also to human–computer interaction (Nass and Moon, 2000), such as gender categorization (Perugia et al., 2022; Roesler et al., 2022) or the use of forms of politeness (Liu et al., 2013; Salem et al., 2014; Babel et al., 2022). There are first studies that have investigated social facilitation in HRI (e.g., Woods et al., 2005; Riether et al., 2012; Wechsung et al., 2014; Hertz and Wiese, 2017). For example, Riether et al. (2012) compared task performance on simple and complex cognitive and motor tasks between individuals working alone or in the presence of a human or a robot. The results showed significant evidence for the predicted social-facilitation effects for both human and robot presence compared to an alone condition. This research shows that typical social effects of human groups can indeed occur in HRI as well.

However, in addition to these positive team effects, there can also be losses for teams. A well-studied phenomenon in human teams is social loafing (Latané et al., 1979; Harkins and Szymanski, 1989; Comer, 1995). It is defined as a lower individual effort on a task performed in a team than on a task performed alone (Karau and Williams, 1993). It has been found that this lower effort is not only a consequence of insufficient team coordination, but also of a change in motivation in shared task settings (Steiner, 1972; Ingham et al., 1974). Social loafing is strongly associated with a lower identifiability of individual contributions and reduced evaluation potential in teamwork, leading to a reduction in motivation (Karau and Williams, 1993). This effect is further moderated by factors such as task valence, coworker performance expectations, and uniqueness of individual task contributions (Karau and Williams, 1993). Specifically, social loafing is higher when the evaluation potential is low, when the task has low perceived value, when a coworker performs well on the task, and when task inputs of the group members are redundant. Social loafing in human teams occurs across different task types and group sizes—even in small teams consisting of only two people (Cymek, 2018; Cymek and Manzey, 2022). For example, in a study by Cymek and Manzey (2022), social loafing was found when two people double-checked the quality of chemical products one after the other. When individuals in the second position in the quality check experienced that the first person was working almost error-free, they checked the quality less often over time and therefore missed more undetected defects than individuals who did the quality check alone. This was expected because the individual performance of the preceding team partner was transparent to the person conducting the checks in the second position, so that the latter’s effort, which is difficult to decipher from the team’s performance anyway, provided only incremental benefit to task completion, thus reducing motivation.

The question of whether this tendency to withhold effort during a collective task with shared output is also relevant to HRI has not yet received much attention. Of course, social loafing may not occur in all forms of HRI. Schmidtler et al. (2015) distinguished three interaction classes of task-related HRI based on working time, workspace, aim, and contact. Coexistence incorporates only a minimum of proximity and dependency. It is characterized by overlapping working time and workspace of the human and the robot. In such a scenario, social-loafing effects should not occur because there is no shared task. Cooperation, in contrast, is additionally characterized by the same aim. Although both parties do not directly depend on each other because of a strict task allocation between humans and robots, the completion of the task by both parties is necessary to achieve the common aim. However, if the outcome of the task is not directly attributable to a particular group member, then social loafing becomes likely. The same applies to collaboration scenarios where humans and robots share the same subgoals and overall goals. When collaborating, both parties are dependent on each other’s actions and work together to achieve a common task, which again opens up the potential for social loafing (Onnasch and Roesler, 2021).

Onnasch and Panayotidis (2020) have already investigated social-loafing effects in HRI. In this laboratory study, participants performed a speed-accuracy task once alone (while the robot also performed the task separately on its own) and once in cooperation with a human or a robotic team partner. Specifically, participants had to place a certain number and color of cotton balls in a gift bag and then place them in a collection box (which was a shared box in the team settings). According to Nass et al. (1996), this manipulation should be sufficient to induce team building in the team conditions, as a simple but credible clarification of whether one was working alone or together was provided (identity) and as team partners were informed that they were working towards a common outcome and would be evaluated together (interdependence). The authors hypothesized effects of social loafing in both team conditions, i.e., the collective human–human condition and the collective human–robot condition, compared to the alone condition. Furthermore, they assumed that social loafing would be more pronounced in the human–robot condition than in the human–human condition due to a reduced sense of being judged or a pressure to justify their performance level when working with a robot compared to a human partner (lower evaluation potential). While there were no differences in performance between the individual and teamwork conditions for either group in the objective performance data (number of filled bags per six-minute trial and number of incorrect filled bags), the subjective data showed a trend in the hypothesized direction. That is, participants in the robot-teamwork condition subjectively reported exerting the least effort compared to participants working with a human or in the solo condition. The authors suggested that the lack of objective social loafing could be due to insufficiently sensitive performance variables or to a low salience of the team setting.

In the current study, we aimed to further investigate the question of the occurrence of social-loafing effects in human–robot teams. While social loafing in redundant quality control has already been demonstrated in humans (Cymek and Manzey, 2022), we wanted to know whether we would also find social-loafing effects in a quality-inspection task performed by a human–robot team, similar to the one described above for electronic components. If social loafing occurs in such a setting, the expected improvement in outcomes due to the redundant quality inspection may not materialize. In our laboratory study, we compared individuals who performed a quality inspection on circuit boards alone with individuals who processed them in a team with the industrial robotic arm Panda. In the latter condition, people performed the quality inspection after the robot and received the usually correct inspection results from the robot. In order to complete the task, participants had to inspect the circuit boards very accurately for defined defects. We hypothesized that the amount of effort that people put into the quality inspection, in terms of the area of the board they searched and the time they spent searching, would be less when working with the robot than when working alone. This reduced effort, if present, should also be likely to have a direct effect on the detection rate of circuit-board defects, which is why the performance of individuals working in teams with the robot should be worse than that of individuals working alone. Since the individuals working in a team with the robot experienced that the robot made few errors (expectation of high co-worker performance), we assumed that the effort invested should decrease over time due to the low cost-benefit ratio. The study was preregistered on the Open Science Framework and the data are available there (https://osf.io/njz2x/).

## 2 Materials and methods

### 2.1 Participants

A total of n = 44 people participated in the study. Based on a G*Power calculation (Faul et al., 2009), the sample size chosen should be sufficient to detect large between-subjects effects and moderate within-subjects and interaction effects in our ANOVAs (α err prob = 0.05, 1-β err prob = .95). However, two participants from the team condition had to be excluded from the data analyses based on prespecified criteria. One did not meet the inclusion criteria because he regularly worked with electronic workpieces, and another marked each robot mark on a circuit-board defect with another mark while not detecting any robot misses, indicating that she did not understand the experimental task. Thus, the final sample included in the data analyses consisted of n = 42 participants. Of these 42 participants, 21 identified themselves as female and 21 as male. All participants were students, had (corrected-to) good vision, spoke German at native-speaker level, and ranged in age from 22 to 30 years (M = 25.55, SD = 2.12). Participants were compensated with course credits.

### 2.2 Task

**FIGURE 1:**
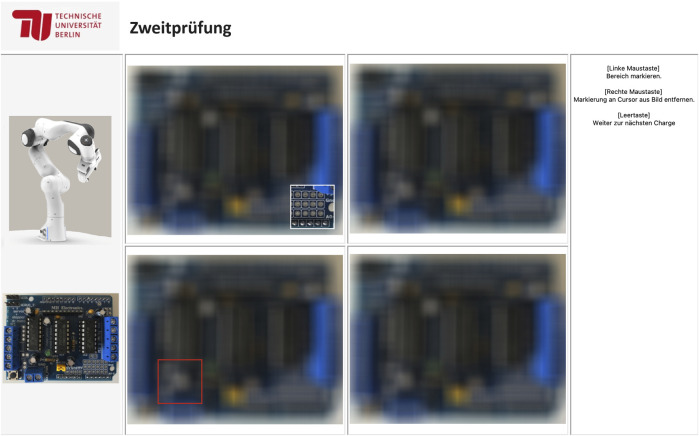
Experimental environment in the team condition. The white square represents the participants mouse while the red square represents a potential error marked by the robot. In the alone condition, the photo of the robot on the left is missing, the header says “Quality Control”, and the images appear without any red mark.

**FIGURE 2:**
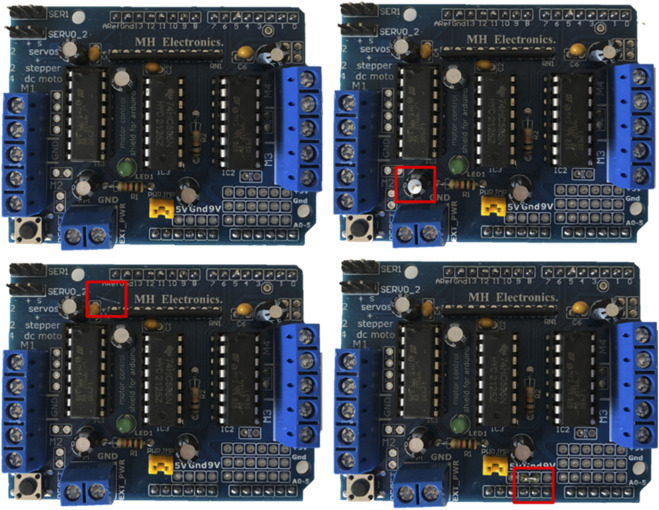
Overview of the error types. Top left: no error; top right: capacitator error; bottom left: scratch; bottom right: soldering error.

Subjects completed a visual-search task that simulated the quality control of circuit boards. Figure 1 shows the user interface of the experimental program. In the center, sets of four circuit-board images were displayed at a time. Each of them contained no, one, or two defects. There were defect capacitors, indicated by a crack in the top of the capacitor, surface scratches, which could potentially affect functioning, and soldering faults, which could potentially lead to short circuits (see Figure 2). The task was to find all of these defects. The images of the circuit boards were initially blurred. To judge the images, participants had to reveal parts of the circuit board step by step. This was done by moving a small, white-framed square over the images with the mouse. Only the area within the moving square was sharp and could be evaluated. Participants were told that the “sharpening tool” would help them to focus during their visual search. This mouse-over approach made it possible to capture search behavior and to track how much of the stimulus participants uncovered. The size of the square was set to 20% of the image width. On the right side of the user interface, software functions such as setting a mark (left mouse click), removing a mark (right mouse click), and proceeding to new images (space bar) were displayed as reminders. On the left side of the board matrix, a reference circuit board without defects was displayed. The user interface varied slightly depending on the condition (team vs. alone). In the team condition, participants worked sequentially redundant with a robot that checked the boards first and set red marks around potential defects (see Figure 1, bottom-left quadrant). In the alone condition, participants worked in parallel, but independently of Panda, on different sets of circuit boards and saw no marks. Also, in the team condition, participants read the header “Double-Check”, whereas in the alone condition the header said “Quality Control”. Last but not least, a picture of Panda was displayed on the left side in the team condition, which was absent in the alone condition.

### 2.3 Design

The experiment used a 2 (condition) x 4 (block) mixed design. The first factor was varied between subjects and included two different conditions: either participants worked alone (while Panda worked simultaneously on different sets of circuit boards) or in the second position in sequential redundancy with Panda (where Panda worked at the first position and checked the circuit boards first). The second factor block was varied within subjects to investigate whether checking effort and/or possible social-loafing effects were influenced by time on task. All participants saw the same 320 images of scanned circuit boards. These were presented to the participants in four blocks of 80 images each. Each block contained 24 randomly distributed defects. In each block, three images contained two defects and 18 images contained one defect. Participants in the team setting saw all the defects correctly marked by Panda in the first three blocks, but could detect five misses of Panda in the failure block #4. The design is summarized in Table 1. In total, Panda detected 94.8% of the defects correctly during the experiment. Participants that worked alone on the task (with Panda working coactively but independently) did not see any defect marks in any of the four blocks.

**TABLE 1:** Number of defects on circuit boards in each group with correctly marked defects (bold) and unmarked defects (!) by the robot in each block.

Condition block	1	2	3	4
Alone	24	24	24	24
Team	**24**	**24**	**24**	**19** & 5!

### 2.4 Dependent variables

We defined four dependent variables: uncovered area, search time, detection performance, and subjective measures (see Table 2). The uncovered area is defined as the average percentage of image area revealed on each board per block. Search time is defined as the average time spent to examine each board with the computer mouse per block. Both variables (e.g., uncovered area and search time) are measures of objective task effort. Detection performance was operationalized as the performance in the five trials in which the robot missed defects in the last block. In addition, four subjective variables were measured with a survey that participants had to fill in after completing the task. It collected subjective ratings on a 7-point Likert scale ranging from strongly disagree to strongly agree. Specifically, participants were asked to rate how much they agreed with statements such as “I put a lot of effort into the visual search.” and “I made a little less effort in the course of the search task.” to learn about the perceived effort and effort over time. The third item measured subjective performance (“I did a very good job on the search task.”) and the final item measured subjective responsibility for the task (“I felt responsible for the task.”).

**TABLE 2:** Operationalization of dependent variables.

Dependent variable	Description
Uncovered area	Average percentage of image area revealed on each board per block via the computer mouse
Search time	Average time spent to examine each board with the computer mouse per block
Detection performance	Detection performance was operationalized as the performance in the five trials in which the robot missed defects in the last block
Subjective measures	Subjective rating on a 7-point Likert scale ranging from strongly disagree to strongly agree with the statements:
	“I put a lot of effort into the visual search.”
	“I made a little less effort in the course of the search task.”
	“I did a very good job on the search task.”
	“I felt responsible for the task.”

### 2.5 Procedure

The procedure is described in Table 3.

**TABLE 3:** Procedure.

	Description
Study invitation	Participants were recruited from a university participant pool. Two separate studies were registered: a “Human–robot-collaboration study” (team condition) and a “Visual-inspection study” (alone condition). This was done so that people knew in advance whether or not they would be working with a robot or not.
Entrance	On entering the room, participants walked past Panda’s workstation and sat down at a computer workstation that was visually separated from the robot by a partition.
Informed consent	Participants in each condition were informed about the experimental setting and their task, the procedure of the test session, and how the data would be kept anonymous. They then gave their informed consent.
Demographics	A short questionnaire asked for basic demographic information (age, sex, vision).
Group manipulation	Participants were briefly told that they would be inspecting circuit boards for defects and whether they would be working in a team with Panda or alone. In the team condition, participants were told that Panda’s results would be forwarded to them for a double check and that they would need to find missed defects or deselect incorrect marks placed by the robot if necessary to achieve the best possible team result. In the alone condition, participants were told that they would be inspecting another set of circuit boards independently of the robot and that they had to find as many circuit-board defects as possible.
Panda demonstration and robot workspace	Panda was then demonstrated in both conditions. The experimenter briefly showed Panda’s workstation and participants watched as the robot, holding a webcam in its gripper, (presumably) photographed and inspected a set of nine circuit boards placed on a tray in front of it. The robot moved from one board to the next, pausing about 10 cm above each one, pretending to take a picture of it. After inspecting the last board on a nine-board tray, the experimenter provided the next tray and the robot moved back to the first board position to begin inspecting the new tray. Two boxes were placed next to Panda, one of which, according to the label, contained “new” circuit boards that would be placed in front of Panda during the experiment to be analyzed, and the other of which, according to the label, would be filled with the “inspected” circuit boards. In addition, a cable connected the robot to the computer the participant was working on, to make the connection between the two workstations seem more plausible.
Written illustrated instructions	The participants read the illustrated instructions to familiarize themselves with the different types of defects. They received a printout of a correct circuit board and of three circuit boards showing the different types of defects. This printout was given to the participants to use it as a reference during the task.
Training	Participants practiced the task briefly. When the participants started training, the robot already started working on the task to get a head start. Thus, participants in the team condition did not have to wait for the inspected boards when they later started the experimental blocks. The experimenter stood next to the robot to supply it with new trays of circuit boards. The continuous supply could be heard but not seen by the participants.
Comprehension check	After the training block, participants had to find and mark one of each defect type on a printout to show that they understood the task.
Experiment	Once the experiment started, participants worked on the task for about 90 min without any feedback on their performance. However, the robot only took 30 min to scan all 320 circuit boards and was switched off at the end. After each experimental block, participants were required to take a short break of at least 1 minute to relax their eyes.
Post-task questionnaire	After completing the task, participants completed a post-test survey.
Debriefing	Finally, they were debriefed and told thank you and goodbye.

## 3 Results

### 3.1 Uncovered area

**FIGURE 3:**
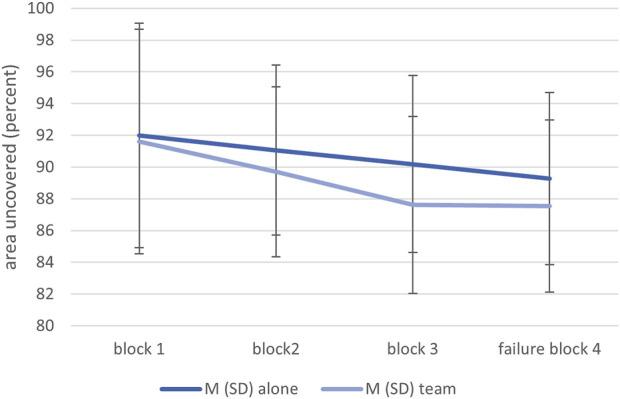
Means and standard deviations of the uncovered area in both conditions and across the four blocks.

On average, a large proportion of the images were searched in both groups and across the blocks. The mean percentage of uncovered area varied within a narrow range of 87.5%–92.0%. A 2 × 3 ANOVA was calculated for the percentage of uncovered area (excluding failure block #4). A highly significant block effect emerged, *F* (1.29, 51.63) *= 12*.66, *p < .*001, *η*_*p*_^*2*^ *=* .24, as all participants searched a smaller area with increasing time on task. No effect was found for the factor condition, *F* (1, 40) *= 0*.74, *p = .395*, *η*_*p*_^*2*^ *=* .02. As can be seen in Figure 3, participants working with Panda in a team checked a slightly smaller proportion of the images descriptively over time compared to the alone condition. However, the interaction effect of block and condition was not significant, *F* (1.29, 51.63) *= 1*.84, *p = .180*, *η*_*p*_^*2*^ *=* .04.

### 3.2 Search time

**FIGURE 4:**
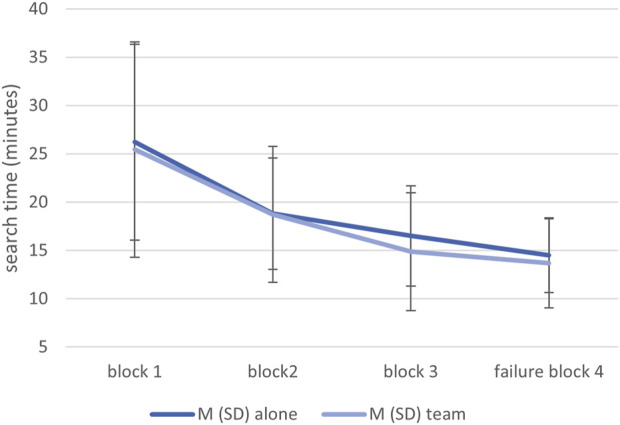
Means and standard deviations of the search time in both conditions and across the four blocks.

A further 2 × 3 ANOVA was calculated to analyze the time spent to search the images. Again, a highly significant effect of the factor block was found, *F* (1.17, 46.62) *= 65*.96, *p < .*001, *η*_*p*_^*2*^ *=* .62. No significant effect of the factor condition was found, *F* (1, 40) *= 0*.14, *p = .708*, *η*_*p*_^*2*^ *<* .01. The interaction was also not significant, *F* (1.17, 46.62) *= 0*.37, *p = .578*, *η*_*p*_^*2*^ *=* .01. Figure 4 shows that mean search time decreased across the blocks but was at the same level in both conditions. Participants took approximately 25 min to search the first block of 80 circuit board images (approximately 19 s per image), 20 min for the second block, and 15 min for the third and fourth blocks (approximately 11 s per image).

### 3.3 Detection performance

**FIGURE 5:**
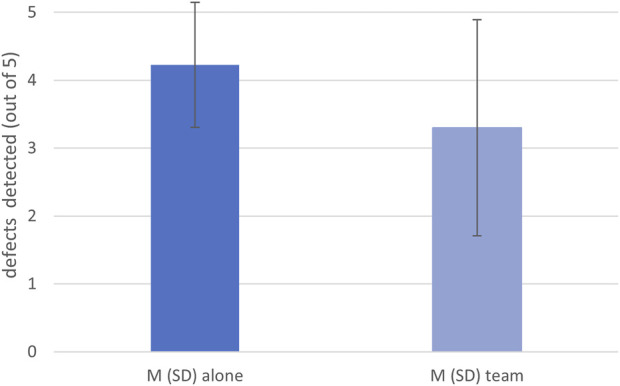
Means and standard deviations of detected defects in block #4 in both conditions.

In block #4, participants in the team condition could potentially miss five defects that were not marked by Panda. Correct detections out of these five potential defects were compared between the two conditions. In the alone condition, the mean detection rate was *M* = 4.23 (*SD* = 0.92), while in the team condition it was *M* = 3.30 (*SD* = 1.59) (see Figure 5). Due to non-normal data and unequal variance, a U-test was calculated. The results indicated that participants working in a team with Panda detected significantly fewer defects than participants working alone, *U =* 148.5, *Z = -1.83*, *p = .029*, *r =* .292.

Note that the people working alone also detected 80% of the defects over the whole experiment (*M* = 19.27 out of 96). The proportion of detected defects is thus comparable between the five trials and the detection performance in the overall experiment for the participants working alone.

### 3.4 Subjective measures

**FIGURE 6:**
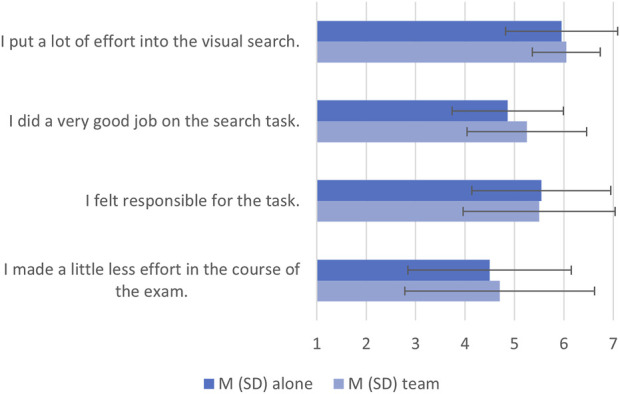
Means and standard deviations of subjective ratings in both conditions on a 7-point Likert scale ranging from strongly disagree (1) to strongly agree (7).

Simple t-tests were performed on the ratings of each statement. No significant differences were found, all *p > .14*. Figure 6 shows that participants in both conditions strongly agreed that they put a lot of effort into the visual search task, and that both groups thought they did a very good job on the task. They also confirmed that they felt responsible for the task and showed moderate agreement with the subjective reduction of effort over time.

## 4 Discussion

As interactions with robots increase, it is important to understand and predict the consequences of human interactions with them. Research on social facilitation has already shown that team processes that occur in human teams can be transferred to human–robot interactions and should be taken into account. The present study investigated whether working with a robot partner would lead to social-loafing effects. Therefore, an experiment was conducted in which participants worked either alone or in a team with a robot on a realistic quality inspection task. Our assumption was that the amount of effort people put into the quality inspection, i.e., the area of the board they searched and/or the amount of time they spent searching, would be lower when working together with the robot than when working alone on the quality inspection, similar to findings of redundant quality control in human teams (Cymek and Manzey, 2022). We also assumed that the individuals working in a team with the robot would reduce their effort over time more than the individuals working alone. In case of a more pronounced effort reduction in the team condition, we assumed that this could lead to a lower defect-detection performance of this group.

There were no group differences in the amount of effort invested in the task for any of the objective measures of effort (i.e., uncovered area, search time). At first sight, this suggests that social loafing did not occur in our experiment. Participants in both groups inspected almost the entire surface of the boards and took their time searching. Over the course of the experiment, participants in both conditions uncovered significantly less image area and accelerated their search. The small decrease in uncovered area may be due to learning that there were some areas of the board where defects did not occur. The large decrease in search time can also be explained by a learning effect. In general, the subjects spent a lot of time searching. In the beginning, they looked at a single image for an average of 19 s, which is a very long time. With more practice they became much faster, but still invested about 11 s per image.

The subjective measures of effort were consistent with the objective measures. Participants in both groups reported that they put a lot of effort into the task, that they felt responsible for the task, and that they performed well. In addition, both groups neither agreed nor disagreed with the statement “I made a little less effort in the course of the search task”, suggesting that participants were aware that they were speeding up their search as time on task increased but were still quite engaged in the task.

We assumed that a reduction in effort might have an effect on the defect-detection performance. Apparently, we found no differences in our effort measures. However, when we compared detection performance on the five common occasions to miss a defect (the five defects in block #4 that were not marked by Panda in the team condition), we found a significant effect. Participants working alone detected on average M = 4.23 (SD = 0.92) of these five defects, whereas in the team condition on average a defect less was detected (M = 3.30, SD = 1.59). There could be several reasons for this disassociation of effort and performance measures. First, it could be that the search speed was too fast to detect the defects. However, this is unlikely as participants in the alone condition searched at a similar speed and found most defects during the experiment (approx. 80% of defects). It could also be that after experiencing a 100% reliable robot for the first three-quarters of the experimental session, participants in the team condition became less suspicious during their search in the last block. It seems as if the participants continued their search routine on the images, as they continued to look at almost the entire circuit board surface. However, they seem to have looked for defects less attentively than the participants who worked alone on the quality inspection.

In the light of these results, we need to consider a phenomenon from a study on cooperation with an automated assistance system. In this study by Manzey et al. (2012), people sampled the information necessary to detect an error, but still did not find it. They also had no idea what the information that had been uncovered actually was. The authors explained this by saying that people looked at the information but did not really process it consciously—in other words, they performed a kind of “inattentive processing” in cooperation with an assistance system. Similar effects have been found in pilots monitoring flight modes in the cockpit. In a study by Sarter et al. (2007), most pilots scanned the mode-annunciator display, but still failed to notice the inappropriateness of the active mode for the current flight context. The authors concluded that the experienced pilots did not process the mode changes thoroughly enough to understand their impact on the behavior of the aircraft. This kind of looking-but-not-seeing effect could have occurred in our experiment as well. Looking but not seeing is characterized by a lower mental engagement and less attentive processing of sampled information. The participants in our study seemed to have maintained the motor routine of uncovering the images with the mouse at a speed that increased slightly over time. So, the motoric effort did not change, the time spent did also not change between the groups, but it seems that the search was carried out with less mental effort and with less attention to the information being sampled. This kind of mental effort is harder to detect but could be measured in future studies using EEG measures such as the mental-engagement index used by Pope et al. (1995).

While Onnasch and Panayotidis (2020) found a tendency for subjective effort to be lower in human–robot teams, this study found lower defect-detection performance when working in a team with a robot. It seems that social loafing is a topic that deserves further investigation. However, as with human teams, it is not always easy to detect motivational losses in teams, such as social loafing, in a laboratory context (Price, 1993), as participants assume that their behavior is being observed and analyzed. Field studies could be an option to find larger effects and get a clearer picture of the impact of social loafing in HRI. It may be that social loafing is more subtle in the lab than in real life and that effect sizes are smaller in the lab. We therefore suggest that future studies try to use a larger sample. In addition, future studies should attempt to replicate our findings while trying to measure the mental effort involved in processing the sampled information.

Our study has several limitations. First of all, we chose an experimental setting that was unlikely to elicit very high levels of group feeling, as participants worked with Panda while visually separated by a partition wall and without the need for communication or direct interaction with the robot. However, participants were told that they would be working in a team, saw the robot as it (presumably) inspected a set of circuit boards before they started their own work on the task, heard the robot’s movements as they worked, had a picture of the robot displayed on their monitor, and saw the marks it (presumably) made, thus constantly reminding participants of the teamwork. Future studies should directly measure the perception of working in a team (e.g., as in Nass et al., 1996) and could investigate the occurrence of social loafing in low, moderate, and high team-perception settings.

Second, social-loafing effects are more difficult to detect when participants are highly aroused (Price, 1993) or when they feel that their individual performance is being evaluated (Karau and Williams, 1993). It is difficult to avoid this completely in a laboratory experiment. Participants need to feel comfortable, well informed, and guided throughout the experiment in order to relax during the test session. Interacting with a friendly and patient experimenter, reading the written instructions at their own pace, and having the opportunity to practice and ask questions should have all helped to reduce participants arousal a bit. In order to reduce the feeling of being evaluated, we chose a set-up where the experimenter could not see the participants while they worked. Also, we did not use eye-tracking, but a more subtle way of measuring where and for how long attention is distributed using our mouse-over approach.

Third, in our experiment, Panda did not actually inspect the circuit boards. To do this, Panda would have needed to be equipped with some kind of vision-analysis software—perhaps based on machine learning—to classify the visual input. Machine learning, such as deep neural networks, are algorithms that can detect patterns they have previously been trained on. We believe that deep neural networks might be well suited to detect production errors on circuit boards. In our setting, we have just claimed that Panda can not only scan the boards but also analyze them for specific defects. Our participants, who all had a human-factors background, did not express any doubts. Although the visual-search task we used seems suitable for machine-learning applications, we chose to work with an embodied robot team partner. We did so because robots are usually perceived more as social agents due to their physicality, and various “social effects” have already been found here (e.g., Woods et al., 2005; Riether et al., 2012). Therefore, we assume that if there is social loafing in human–machine interaction, it should be particularly the case for embodied and autonomous agents. Future studies should investigate social loafing in interaction with non-embodied AI, as the effects could in principle also be conceivable here.

Robots are becoming increasingly important in many industries and can take over more and more tasks. However, they are often not yet capable of working fully autonomously and without supervision. For this reason, in many industries and for many tasks, human supervision or augmentation of the robot’s work will be required for some time to come. Combining the capabilities of humans and robots obviously offers many opportunities, but we should also consider unintended group effects that might occur in human–robot teams. When humans and robots work redundantly on a task, this can lead to motivational losses for the human team partner and make effects such as social loafing more likely. Social loafing should therefore be taken into account.

## Data Availability

The datasets presented in this study can be found in online repositories. The names of the repository/repositories and accession number(s) can be found below: https://osf.io/njz2x/.
